# Turning antibodies off and on again using a covalently tethered blocking peptide

**DOI:** 10.1038/s42003-022-04094-1

**Published:** 2022-12-10

**Authors:** Michael Brasino, Eli Wagnell, Sean Hamilton, Srivathsan Ranganathan, Michelle M. Gomes, Bruce Branchaud, Bradley Messmer, Stuart D. Ibsen

**Affiliations:** 1 Cancer Early Detection Advanced Research Center, Knight Cancer Institute, Oregon Health and Science University, Portland, OR 97201 USA; 2 Department of Biomedical Engineering, School of Medicine, Oregon Health and Science University, Portland, OR 97201 USA; 3 Abreos Biosciences, San Diego, CA 92122 USA

**Keywords:** Protein design, Protein design, Cell delivery, Antibody therapy

## Abstract

In their natural form, antibodies are always in an “on-state” and are capable of binding to their targets. This leads to undesirable interactions in a wide range of therapeutic, analytical, and synthetic applications. Modulating binding kinetics of antibodies to turn them from an “off-state” to an “on-state” with temporal and spatial control can address this. Here we demonstrate a method to modulate binding activity of antibodies in a predictable and reproducible way. We designed a blocking construct that uses both covalent and non-covalent interactions with the antibody. The construct consisted of a Protein L protein attached to a flexible linker ending in a blocking-peptide designed to interact with the antibody binding site. A mutant Protein L was developed to enable photo-triggered covalent crosslinking to the antibody at a specific location. The covalent bond anchored the linker and blocking peptide to the antibody light chain keeping the blocking peptide close to the antibody binding site. This effectively put the antibody into an “off-state”. We demonstrate that protease-cleavable and photocleavable moieties in the tether enable controlled antibody activation to the “on-state” for anti-FLAG and cetuximab antibodies. Protein L can bind a range of antibodies used therapeutically and in research for wide applicability.

## Abstract (web summary)

Modulation of antibody binding is developed through a photo-cleavable tether that is covalently conjugated to the antibody and to a blocking peptide.

## Introduction

Monoclonal antibody technology has revolutionized biotechnology and medicine. Despite the development of multiple alternative binding proteins, such as nanobodies, affibodies and alpha bodies, antibodies are still the most widely used affinity agent both in the lab and for therapeutic applications^1,2^. Antibodies are naturally in an “on-state” in the sense that they are always capable of binding to their targets and this capacity cannot be temporally or spatially controlled under physiological conditions. For cancer immunotherapy applications where the antibodies induce immune activation by blocking regulatory signaling proteins on the surface of immune cells, this creates a challenge^3^. The infused therapeutic antibodies are always in an “on-state” causing the antibodies to bind not just to immune cells in the region where they would be the most effective, such as tumor draining lymph nodes^4^, but also throughout the body, resulting in life threatening side effects^5^. The ability to control antibody binding activity, such as changing the antibody from a nonbinding “off-state” to a binding “on-state” with spatial and temporal control is critical for localizing antibody binding in therapeutic applications and in many other biological applications and assays^6,7^.

The complex structure of monoclonal antibodies has historically complicated their structural modification and functionalization. The idea of synthesizing activatable antibodies has been investigated previously, but past approaches have had challenges^8–13^, which our design addresses. For example, protease activated antibodies were recently realized through the genetic fusion of two interacting capping peptides at the N-termini of both the antibody heavy and light chains^14^. The resulting antibody’s binding was blocked until a protease cleaved these capping peptides, allowing the antibody to activate and bind its target. This capping strategy called for the genetic modification of the antibody sequence, requiring monoclonal antibody expression outside the means of most laboratories. Here, we demonstrate that a capping-based strategy can be accomplished using a site-specific conjugation method without the re-expression of an antibody.

Currently, antibody blocking techniques which specifically block only the antibody binding pocket without needing to modify the primary antibody sequence, are limited. For example, antibody blocking and activation were recently achieved by divalent blocking peptides linked by double stranded DNA^13^. Various modifications allowed light, pH, or protease activity to trigger the cleavage of this DNA, causing a loss of peptide valency, resulting in antibody un-blocking. We sought to further enhance and secure antibody blocking by covalently tethering the blocking peptides to a region just outside the antibody binding pocket. This strategy uniquely takes advantage of simultaneous covalent and noncovalent attachments to the antibody.

We achieved the noncovalent blocking of the antibody binding site using a blocking peptide designed specifically for the antibody. The blocking peptide has a relatively low binding constant when in free form, but has an artificially elevated binding constant when connected to the covalently bound tether which keeps the blocking peptide near the antibody binding site encouraging rebinding after the peptide naturally unbinds. This creates an effectively high local concentration of the peptide at the antibody binding site. This allows the blocking peptide to successfully outcompete the intended antibody target and keep the antibody in an “off-state”. The tether is designed to be cleaved, and once broken, the blocking peptide will naturally unbind and diffuse away due to Brownian motion, preventing it from rebinding. This effectively converts the antibody to an “on-state”. In this way, we are able to provide stable, long-term blocking of the antibody binding site and also enable quick restoration of binding upon activation without making any changes to the structure of the native antibody itself. We can incorporate non-proteinaceous linking sequences into the tethers, which cleave in response to light, thereby creating photoactivated therapeutic antibodies. Protease cleavage can be employed as the activating trigger as well, which demonstrates the activation mechanism can be tailored for specific applications.

The covalent binding of the tether molecule to the antibody was achieved using site-specific conjugation methods. Many site-specific labeling schemes look to achieve site specificity, such as maleimide modification of sulfhydryl groups (cysteine) or N-hydroxy-succinimide modification of amine groups (lysine), but they frequently label each IgG a variable number of times at multiple locations across the four protein chains (two light and two heavy)^15^. To address this, several groups have taken IgG binding proteins, such as Protein G and A, which bind at specific locations on the heavy chain of IgG outside of the antigen binding region, and modified them to covalently attach to the IgG at those sites, providing a truly site-specific conjugation handle^16–19^. These have allowed the conjugation of drug payloads for targeted therapy, and dyes or imaging agents for immunostaining. However, these attachment sites are not located near the antigen binding site of the antibody as would be ideal to attach tethered blocking peptides. More recently, Protein M, which does bind the light chain, has been investigated for controllable antibody blocking, but without further engineering to enable covalent attachment^20^. Therefore, we have taken the well-studied protein L, which binds in close proximity to the antigen binding site of most immunoglobulins, and engineered it to attach covalently.

## Results

### Design of an antibody tethered blocking peptide using PpL

**Fig. 1:**
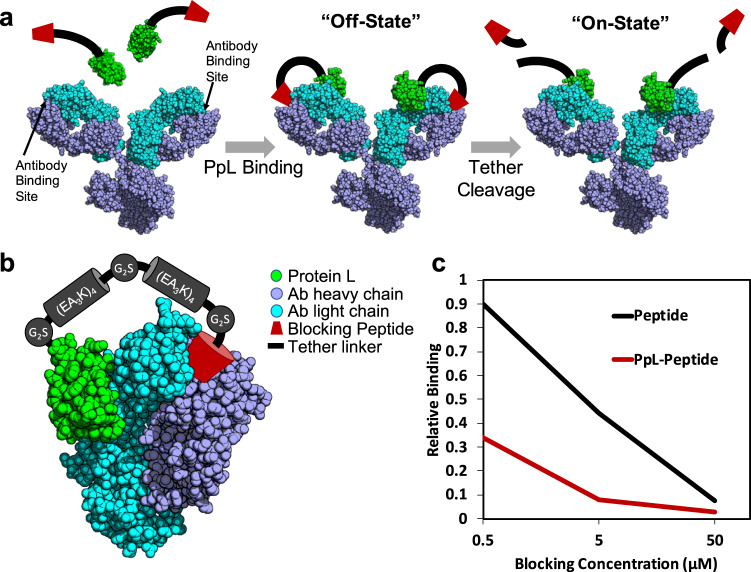
Antibody inactivation through PpL based attachment of a tethered blocking peptide. **a** Schematic of the antibody blocking and activation strategy. **b** Rendering of the crystal structure (PDB 1MHH)^21^ of PpL (green) bound to a Fab fragment of an IgG molecule. The path of a proposed linker is shown in cartoon form with the different segments labeled with their sequence. It is attached to the C-terminus of PpL (green) and reaches up and across the antigen binding pocket between the light (cyan) and heavy (blue) chains. In order to bend, the linker is broken into two alpha helical segments made to be approximately 3 nm each. **c** PpL linked to the flag epitope was shown to block an anti-FLAG antibody better than the flag epitope alone. Relative binding was estimated by ELISA and is expressed in relation to the anti-FLAG antibody alone. (*n* = 1, graph for illustrative purposes).

In order to keep the blocking peptide near the antibody’s binding pocket we aimed to attach the tether to the native antibody itself as shown in Fig. 1a. To site specifically attach this tether to the antibody, we used Protein L, due to its well-documented site-specific binding to a majority of human (and mouse) antibodies. Protein L from *Peptostreptococcus magnus* contains several repeated B domains that bind to most subtypes of the kappa (κ) light chain without interfering with antigen recognition. Unlike similar antibody binding proteins, these B domains only bind the light chain, and have no affinity for the heavy chain or fragment crystallizable (Fc) region, the latter of which mediates the function of immunotherapeutic antibodies. For our purposes, we used a modified version of a single B domain (referred to as PpL). We estimated the distance between the C-terminus of PpL and the binding pocket of a bound antibody to be approximately 7 nm, based on published structures^21^. Also apparent was the need for this linker to be flexible and to reach over the lip of the antigen binding pocket of many antibodies. To satisfy both these requirements, we created a protein linker composed of synthetic alpha helices for appropriate length^22^, separated by short stretches of glycine-serine sequences for flexibility (Fig. 1b). Initial experiments using this PpL-linker-peptide construct demonstrated that both a blocking peptide (the FLAG sequence DYKDDDDK) and PpL worked together to block an anti-FLAG antibody (clone 1557CT661.18.1), helping validate our linker design (Fig. 1c). However, we predicted we could increase the blocking efficiency further by covalently binding PpL to the antibody, thereby keeping the blocking peptide tethered permanently to the antibody and keeping the antibody in the “off-state” until the tether was cleaved.

### Covalent attachment of tether to antibody through a photoconjugated PpL

**Fig. 2:**
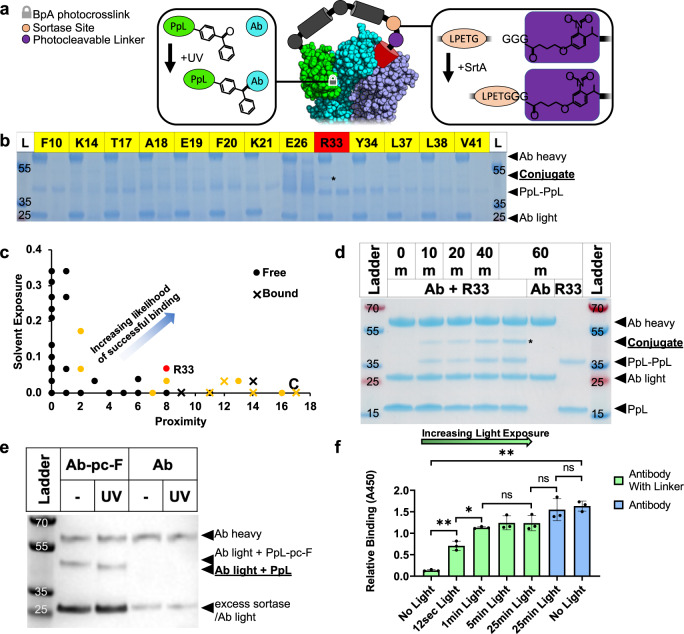
Successful photoconjugation of PpL to an antibody light chain, and successful blocking of an anti-FLAG antibody followed by light activation. **a** Different locations were chosen on PpL based on the crystal structure (PDB 1 mhh) to introduce BpA. **b** A reducing SDS-PAGE gel with 50 µM of each PpL mutant irradiated with 1 µM mouse anti-CD3 antibody shows a photoconjugated product between the light chain and PpL (*) only with the R33BpA location, shown in red. Other tested locations are shown in yellow. All gels are labeled with a mass ladder control in kDa. **c** Graph showing each amino acid in PpL with the solvent exposure level and number of antibody carbons that are within 1 nm. The higher the solvent exposure and the higher the number of proximal carbons the more likely the amino acid was to be a successful candidate for modification with BpA. Yellow shows which amino acids were modified and red shows amino acid R33 which was ultimately successful with a high surface exposure and number of proximal carbons. The amino acids are also labeled as “Free” or “Bound”, based on a 0.35 nm distance cut-off between the PpL sidechain and antibody atoms. **d** Reducing SDS-PAGE gel showing 100 µM of PpL-R33BpA (R33) with 4 µM mouse anti-CD3 antibody (Ab) irradiated under 360 nm light for the time indicated. PpL-R33BpA photoconjugated to the light chain is indicated with (*). **e** The R33BpA mutant with the linker arm discussed above was photoconjugated to the anti-FLAG antibody and then attached via sortase to a photocleavable blocking peptide. Light exposure for 10 min lead to photocleavage and loss of the blocking peptide. **f** The anti-FLAG antibody alone or modified with the photocleavable blocking peptide (as in **e**), was treated with 365 nm light for the indicated time, then diluted to 10 nM and analyzed via ELISA for binding (*n* = 3). The tethered blocking peptide successfully reduced the binding efficiency of the anti-FLAG antibody to its target and was removed with brief light exposure, leading to activation of antibody binding. **P < 0.01 and *P < 0.05 by Welch’s *t*-test (two-sided). Bar height shows data average. Error bars are standard deviation.

Covalent attachment of PpL to the antibody was accomplished by modifying the PpL to contain a non-canonical amino acid with a reactive side chain. Using an amber codon suppression technique developed previously^23^, we substituted the photo-crosslinking non-canonical amino acid 4-benzoyl phenylalanine (BpA) at various positions within the predicted binding interface of PpL to determine which site resulted in the best binding efficiency. BpA has been previously used to create site-specific covalent attachments between proteins including proteins G and A to IgG^16,24^, but its use with PpL has not been demonstrated previously. As shown in Fig. 2a–c and S2, we modified the PpL-linker-FLAG construct with BpA at various locations and screened each individual mutant for photoconjugation to a mouse IgG1 kappa anti-CD3 antibody. The use of this antibody, which does not bind the FLAG antigen, precluded any interference due to binding of the blocking peptide to the antibody.

The structure of PpL bound to the antibody was used to predict which amino acids in PpL would be good candidates for modification with the BpA (Fig. 2c). These needed to be amino acids with a high level of surface exposure indicating they were not sequestered in the middle of the protein, as shown by the *y*-axis. More importantly, the amino acid candidate would also need to be proximal to the antibody, as indicated by the number of antibody carbons within a 1 nm observation volume centered at C_alpha_, as shown on the x-axis. In this plot, the farther the amino acid falls from the origin, the better we expect it to perform. However, mutating amino acids that are directly interacting with the antibody (defined by a 0.35 nm distance cut-off) would likely negatively affect PpL binding. Therefore we classified these amino acids as “Bound” and the rest as “Free”, and expected the “Free” amino acids on the surface with a high number of proximal antibody carbons to give the best results. The amino acids that are shown in yellow were modified to determine binding efficiency. R33 (shown in red) was ultimately the successful binding candidate and had a good combination of surface exposure and a high number of proximal antibody carbons.

The specificity of this conjugation was then explored, as shown in Fig. 2d and S3. Photoconjugation occurred between the PpL construct and the anti-CD3 light chain, while no attachment to the heavy chain was observed. PpL dimers were also seen, but both dimers and un-reacted monomers were easily cleaned from the photoconjugated antibody through filtration techniques in later photo-conjugation reactions (see below). This purification did not remove antibodies without PpL conjugation, or with only a single PpL conjugation. Initial yield of conjugated to un-conjugated light chain was relatively low under these conditions (approximately 30% by gel intensity). Yields were increased in subsequent photo-conjugations up to 79% by decreasing the antibody concentration to 2 µM and using freshly purified PpL. PpL binds most subtypes of the kappa light chain and these subtypes are exceedingly common in biological applications, leading to PpL binding more than half of all immunoglobulins in human serum and over a third in mice^25^. As such, this photoconjugating mutant should allow covalent attachment to most antibodies used in the lab and clinic. While not attempted here, this conjugation strategy could theoretically aid in the site-specific conjugation of antibody Fab fragments, for which there are currently few options.

### Blocking and un-blocking of an anti-FLAG antibody

Next, we used this R33 mutant PpL to covalently bind to the anti-FLAG antibody. For this, we were able to saturate the anti-FLAG antibody with PpL by repeatedly purifying the antibody using filtration, adding additional PpL and photoconjugating (see supporting information). Furthermore, our design allowed for reversible blocking of the antibody by attaching an anti-FLAG blocking peptide with an N-terminal photocleavable linker. To avoid cleaving this linker during the photoconjugation reaction, it was attached only after the R33 mutant was photoconjugated to the antibody. For this, we inserted the transamidase Sortase A recognition site (LPETG) at the C-terminus of the linker arm and had the photocleavable blocking peptide synthesized with a tri-glycine motif at its N-terminus. Sortase A was then used to cleave the partial multiple cloning site and His-Tag from the C-terminus of the sortase site and replace it with an amide bond to the N-terminal glycine of the photocleavable synthetic peptide. While this caused only a slight (0.5 kDa) drop in mass that was difficult to detect by gel, light exposure led to the complete photocleavage of the blocking peptide as observed by SDS-PAGE (Fig. 2e and S4**–**5). This complete cleavage in-turn suggests a complete Sortase conversion. Photocleavage corresponded to successful restoration of anti-FLAG binding abilities, while the anti-FLAG antibody itself was unaffected (Fig. 2f). A significant dose dependence was seen on antibody activation up to 1 min of light exposure. There was no significant difference in binding between the activated antibody exposed to 25 min of light and the pure antibody exposed to 25 min of light indicating a high degree of activation was achieved. This demonstrates the ability to temporally control the activation of the anti-FLAG antibody and enables future work to use the localization of light to control the spatial activation of the antibody within a tissue or sample.

### Blocking and un-blocking of the therapeutic antibody cetuximab

**Fig. 3:**
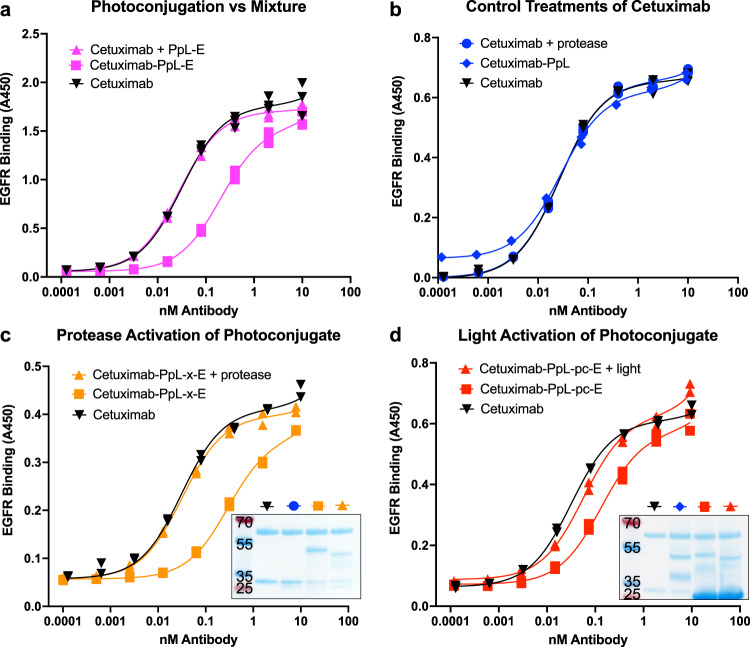
Protease and light activation of therapeutic cetuximab. **a** Cetuximab was mixed with blocking peptide linked to wildtype PpL, which binds the light chain transiently (Cetuximab + PpL-E), or was photoconjugated to blocking peptide linked to the R33BpA mutant PpL, which binds the light chain covalently (Cetuximab-PpL-E). Both were compared for affinity to EGFR vs cetuximab alone (Cetuximab). The addition of two molar excess PpL-E had no noticeable effect on cetuximab affinity indicating the PpL needs to be photoconjugated to effectively block the antibody (K_D_ values – Cetuximab 31 pM, Cetuximab-PpL-E 201 pM, Cetuximab + PpL-E 27pM) (*n* = 3). **b** Protease (chymotrypsin) treatment had no effect on the affinity of cetuximab for EGFR (Cetuximab + protease), nor did the photoconjugation of PpL and the linker arm without blocking peptide (Cetuximab-PpL) (K_D_ values - Cetuximab 26pM, Cetuximab + protease 26 pM, Cetuximab-PpL 28 pM) (*n* = 2). **c** Reducing SDS-PAGE gel and ELISA (*n* = 2) of cetuximab alone versus cetuximab photoconjugated to R33BpA mutant PpL linked to a blocking peptide via a protease cleavable linker (Cetuximab-PpL-x-E), with and without protease treatment. Photoconjugation of the blocking construct lead to a ~9-fold lower affinity than cetuximab alone. Protease treatment resulted in a marked decrease in the molecular weight of the photoconjugated light chain (corresponding to loss of the blocking peptide) and rescued cetuximab affinity (K_D_ values – Cetuximab-PpL-x-E 293 pM, Cetuximab-PpL-x-E + Protease 33 pM, Cetuximab 31 pM). **d** Reducing SDS-PAGE gel and ELISA (*n* = 2) of cetuximab alone versus cetuximab photoconjugated with R33BpA mutant PpL linked to a blocking peptide via a photocleavable linker (Cetuximab-PpL-pc-E), with and without 10 min of 365 nm light exposure. The photoconjugate with blocking peptide had a markedly decreased affinity for EGFR. Light exposure lead to a decrease in molecular weight of the photoconjugated light chain (corresponding to the loss of the blocking peptide), and EGFR affinity being largely restored. (K_D_ values - Cetuximab 31 pM, Cetuximab-PpL-pc-E 131 pM, Cetuximab-PpL-pc-E + light 57 pM) (*n* = 2). All gels labeled with size control ladder bands in kDa.

We then tested this photoconjugation and reversible blocking strategy on the therapeutic antibody cetuximab, which binds to epidermal growth factor receptor (EGFR) and is routinely used in immunotherapy for multiple cancers^26^. For this, we fused a blocking peptide (QGQSGQCISPRGCPDGPYVMY) known to block cetuximab at the C-terminal end of the tether attached to the photoconjugating PpL. This construct was purified from E. coli and immediately photoconjugated at 100 µM to cetuximab (2 µM) in a single pass with 1 h of 365 nm light, then filtered to remove un-bound PpL. The resulting photoconjugate was then tested for affinity to EGFR via ELISA. Each data series for Fig. 3 shows specific binding activity to EGFR and was collected with non-specific binding controls. Non-specific binding was similarly low for all antibody constructs tested. As shown in Fig. 3a, this photoconjugate was found to bind with a 6-fold lower affinity than the un-modified cetuximab. The resulting photoconjugates contained at most two PpL constructs for every antibody, and so a control construct which lacked the R33BpA mutation but contained the same tether and blocking peptide sequence was mixed at a two to one ratio with cetuximab and measured as well. We observed no difference in affinity between this mixture and cetuximab alone. This demonstrates the necessity of covalent attachment through mutant PpL photoconjugation, as the combined avidity of both the blocking peptide and unmutated PpL were insufficient to keep the construct in place and block cetuximab binding. There was also no change in affinity when a construct without blocking peptide was photoconjugated (Fig. 3b). This demonstrates that covalent PpL attachment (even with attached linker sequence but no blocking peptide) did not interfere with cetuximab binding, and suggests this modification strategy will not adversely affect the therapeutic behavior of the antibody once the tether is cleaved.

We then used protease activation to test whether the photoconjugated antibody binding could be restored by removing the blocking peptide. For this, we used chymotrypsin as a model test system protease, and a short chymotrypsin-cleavable peptide sequence (GGSAAPFGG) was inserted between the linker arm and blocking-peptide sequence. This protease-cleavable version was conjugated to cetuximab as before. Figure 3b shows that chymotrypsin exposure did not affect the cetuximab binding activity. SDS-PAGE was used to monitor the successful photoconjugation of the protease-cleavable tether to cetuximab, as well as cleavage of the blocking peptide from the light chain upon chymotrypsin incubation (Fig. 3c, inset and S6). This construct successfully blocked cetuximab from binding EGFR until activation with the protease chymotrypsin, at which point its affinity increased 9-fold as determined by ELISA (Fig. 3c).

Finally, to activate cetuximab with light, we attached the cetuximab blocking peptide to the linker arm via a photocleavable linker (CAS 162827-98-7) using the strategy discussed above for anti-FLAG. Due to the larger weight of the cetuximab vs FLAG blocking peptide, the sortase reaction led to a slight increase (0.7 kDa) in PpL MW. Accordingly, SDS-PAGE analysis (Fig. 3d, inset and S6) indicated the successful attachment of the tethered photocleavable blocking peptide to cetuximab and the release (2.2 kDa) of the peptide upon 365 nm light exposure. Excess Sortase A (17.9 kDa), did not filter completely from the reaction likely due to forming dimers in solution, but these dimers did not interfere with antibody affinity. As shown in Fig. 3d, the attached photocleavable blocking peptide led to a 4-fold decrease in affinity to EGFR. Moreover, light exposure restored cetuximab to its original binding affinity. This 4-fold difference was notably lower than that of the protease cleavable construct which could be partly explained by the differing yields in photoconjugating each construct, as seen in each respective gel. We observed that PpL fused to the blocking peptide before photoconjugation (as in the case of the protease cleavable version) more completely photoconjugated to cetuximab (yield of 79% by gel). In contrast, PpL with the sortase site only (as used in the photocleavable version) gave a lower yield (75% by gel). Photoconjugation could be enhanced by fusing a blocking peptide to the C-terminus of the Sortase site. However, this hindered the Sortase reaction from completely substituting this bound blocking peptide for the synthetic photocleavable version. Additional rounds of photoconjugation and purification might be used to further saturate the antibody light chains, or high resolution size exclusion chromatography may allow the removal of antibodies that do not contain two photoconjugated light chains.

## Discussion

Antibody switches that can be spatially and temporally regulated have widespread applications in both an immunotherapy landscape, where antibodies are widely used, as well as in the laboratory setting. Here, we demonstrate for the first time the successful blocking and controlled unblocking of antibodies by designing a construct that takes advantage of both precise covalent and noncovalent interactions with the native antibody.

Our development of a photoconjugatable version of PpL allows for site specific covalent anchoring of the blocking construct to a known location on the antibody light chain. This location is close enough to the antibody binding site to allow for effective noncovalent interaction with the tethered blocking peptide and far enough away to prevent interference to antibody binding from the PpL molecule itself. This tethering distance allowed the blocking peptide to outcompete the intended target thereby putting the antibody into an “off-state”. Upon cleavage of the tether, via protease or 365 nm light, the non-covalently bound blocking peptide was released and the antibody switched to an “on-state”. Covalently binding the PpL to the antibody allows blocking peptides that have lower affinity for the antibody to successfully outcompete the intended target. The benefit to having a blocking peptide with lower binding affinity is that when the tether molecule is cleaved it will allow the antibody to quickly return to an “on-state” once the freed blocking peptide naturally unbinds and diffuses away due to Brownian motion.

We successfully demonstrated two different activation mechanisms that cleave the tether and activate the antibody. The first was a protease-based cleavage technique using chymotrypsin. The inactivated cetuximab showed a ~9-fold lower affinity than cetuximab alone, but affinity was rescued with protease treatment that cleaved the tether and activated the antibody. The second mechanism used a photocleavable moiety and inactivated cetuximab showed a 4-fold reduction in binding affinity. The binding affinity for EGFR was largely restored upon 365 nm light exposure. Future work will seek to further enhance this blocking effect. This technique was demonstrated using both the anti-FLAG antibody and the therapeutic antibody cetuximab, making the technique promising for future therapeutic applications where it may reduce the side effects of systemically administered active antibody.

The use of light as an activating trigger is particularly important for therapeutic applications. It allows for the use of photocleavable linkers that are resistant to protease based cleavage. This addresses situations where proteases which are overexpressed in tumors^27–29^ are also present in non-target tissues^30,31^, especially in the liver^32,33^, which could result in large scale non-localized activation of the antibody. Light also allows the user to activate the antibody in tissue regions which may not have significant overexpression of tumor-related proteases, such as in the tumor draining lymph nodes, where immunotherapy can be most effective^4,34,35^. Light activation can also be applied to a larger population of patients because it is independent of specific tumor based biochemistry which can have high variability between cancer patients^36^. Importantly, the blocking and un-blocking strategy developed here is particularly well suited to immunotherapies, as it leaves the Fc region (which mediates immune function) un-modified.

The wavelength of light is critical to achieve spatial localization within the body. Here, we have chosen light with a 365 nm wavelength which is effective at triggering photocleavage of our construct and has low absorption by internal tissue^37^ as well as DNA^38^ reducing possible tissue damage from light exposure. This wavelength has been shown to activate photocleavable prodrugs in a 1 cm diameter when delivered to the center of a tumor^39^. The 365 nm light is highly scattered by the tissue^40^ creating a uniform exposure region^39^. One of the benefits of 365 nm is that although it has useful penetration depth through internal tumor tissues, it does not penetrate deeply through skin due to melanin absorption^41^. This prevents uncontrolled activation of the antibody from external light sources. The 365 nm light can be delivered through the skin to the tissue region of interest by fiber optic coupled light emitting diode systems^39^ or through miniaturized light emitting diode technology where elements can be made with submillimeter dimensions^42^ allowing them to be implanted using biopsy needles or laparoscopically. This internal delivery of 365 nm light keeps the activation region localized to just the targeted tissue region. Near infrared wavelengths could be used as an activating trigger through the incorporation of different photocleavable compounds. It has greater penetration depth through skin and tissue and would allow activation over a larger region. The use of near infrared light would cause antibody activation in the skin and in the intervening tissue between the skin and the internal target tissue which may be beneficial in certain therapeutic applications.

In future work, the synthesis and purification process used to produce the antibody conjugates will be further refined. The PpL photoconjugation wavelength was also the wavelength that could cleave the light sensitive tether making the attachment a two-step process. These two steps reduced the overall conjugation yield of the light activated antibody, as demonstrated by the improved blocking of the protease activated antibody, which could be synthesized in a single step. In both versions, the filtration protocol was unable to remove antibodies that were bound to only a single blocking peptide. Future work will look to develop large-scale photoconjugation and purification of fully blocked cetuximab molecules from those with one light chain left un-conjugated. This will produce uniform cetuximab conjugates with much lower affinities before activation with light or protease treatment.

## Methods

### Expression of PpL constructs

A single domain of the multimeric Protein L (PpL) was used for our studies to create a precise anchoring point for our capping peptides. For this, we used the engineered C* domain which is nearly identical to the C4 domain of wildtype Protein L (Uniprot Q51918). This domain forms the same secondary structure as it’s wildtype counterpart and binds the kappa light chain with a dissociation constant of 130 nM^43^. In order to create a long but flexible linker sequence, two alpha helices each composed of four repeats of the synthetic EAAAK sequence were appended to the C-terminus of the PpL domain. For flexibility, these alpha helices were separated from the PpL and each other by short sequences of glycine and serine. This protein sequence was then codon optimized for expression in *E. coli* and synthesized (Integrated DNA Technologies) before being inserted into the pET21b(+) expression vector (EMD Millipore). This was done by amplifying both vector and insert with PCR primers containing compatible 5’ overhangs and then assembling them via the NEB HiFi assembly reaction (New England Biolabs). Proper insertion was confirmed via Sanger sequencing (Genewiz). A complete nucleotide sequence for this expression construct is included in supplemental documents (Fig. S1).

For expression, the BL21 (DE3) strain of *E. coli* (ThermoFisher) was transformed with this plasmid and maintained in 100 µg/mL ampicillin (GoldBio) for selection. Transformants were grown in 5 mL of Luria Broth (LB) overnight at 37 °C, followed by 100 fold dilution into LB the following morning. Once this new culture reached mid-log growth, as indicated by an OD_600_ of approximately 0.4, Isopropyl β-D-1 thiogalactopyranoside (IPTG) was added to a final concentration of 1 mM to induce the expression of the PpL construct. Cultures were allowed to express for 4 h followed by centrifugation at 10,000 g to collect cells and remove culture media. Cells were then lysed by freezing pellets overnight at −20 °C followed by resuspension in 30 mL of equilibration buffer (20 mM Phosphate Buffer at pH7.6, 300 mM NaCl, 10 mM Imidazole), followed by sonication using a probe sonicator (Qsonica, model Q500) with half inch probe diameter for 4 min total with 30 s on/off pulses, 40% amplitude. Insoluble material was then removed through centrifugation at 12,000 g for 20 min. PpL constructs expressed on pET vectors contained a C-terminal 6xHis tag and were purified using immobilized metal affinity chromatography. 50 µL of sedimented Ni-NTA coated agarose beads (ThermoFisher) were added to the soluble fraction and allowed to bind for 1 at 4 °C in an end-over-end mixer. Beads were then removed via centrifugation at 700 g for 2 min and then washed four times with 400 µL wash buffer (20 mM PB, 300 mM NaCl, 25 mM Imidazole). Finally, PpL was eluted from the beads in 200 µL elution buffer (20 mM PB, 300 mM NaCl, 250 mM Imidazole). Proteins were transferred into PBS using 7 kDa MWCO desalting columns (ThermoFisher) and quantified via A280 signal with their predicted extinction coefficient (calculated from amino acid content).

### Mutagenesis of PpL for photoconjugation

To further modify PpL to covalently attach to the kappa light chain, we substituted the non-canonical amino acid *p-*Benzoyl Phenylalanine (BpA) at multiple locations in and around the previously determined binding interface^21^. Fourteen amino acids were initially chosen for substitution. Using PCR mutagenesis (Q5 site-directed mutagenesis kit, New England Biolabs), the codon for each amino acid was mutated to the amber stop codon (TAG) to allow for BpA incorporation via the amber suppression method^44^. pET vectors containing the mutated PpL proteins were then co-transformed into the BL21 (DE3) *E. coli* strain along with the pEVOL-pBpF plasmid (provided by the lab of Peter G. Schultz, Addgene #31190)^23^ which contains both the aaRS and tRNA needed to incorporate BpA at amber codons. The resulting transformants were grown under selection with 100 µg/mL ampicillin and 25 µg/mL chloramphenicol. For expression, transformants were grown overnight in 5 mL of LB at 37 °C followed by 1:100 dilution the following morning. This production culture was typically as little as 50 mL but could be scaled up as necessary. Cultures were grown until mid-log phase (OD_600_ = 0.4) at which point IPTG was added to a 1 mM final concentration to induce mutant PpL expression and arabinose was added to 0.2% (wt/vol) final concentration to induce aaRS and tRNA expression from pEVOL. At the same time BpA was added directly to the cultures for a final concentration of approximately 1 mM. Cultures were allowed to express for 4 h followed by centrifugation at 10,000 g to collect cells and remove culture media. Purification of mutant PpL was performed in the same manner as PpL detailed above.

### Photoconjugating mutant modeling

To predict which amino acids on protein-L could be modified for photoconjugation to the antibody, we utilized the 3D structural model of protein-L bound to the light chain of IgG (1YMH.pdb)^45^. Based on the structure, we performed calculations for each amino acid to determine both the surface exposure and proximity of the amino acid side-chain carbons to the antibody light chain carbons. To determine surface exposure, we calculated the solvent accessible surface area (SASA) of the side-chains using the gmx sasa tool of GROMACS 2018^46,47^. We then determined the number of antibody sidechain carbons in the vicinity (<1 nm) of each amino acid. We also identified the amino acids that were directly involved in interaction with the antibody, using a distance cut-off (<0.35 nm), which were designated as “Bound” in Fig. 2c. These amino acids were likely to be involved with the noncovalent binding of PpL to the antibody and modifications to them would likely negatively affect this binding. We were able to identify the residues that were surface exposed and highly proximal to the antibody without interfering with the antibody/PpL interaction.

### Photoconjugation

In initial screens, PpL mutants and anti-CD3 (clone UTH1, BD bioscience 555329) antibodies were diluted into PBS pH7.6 such that the final concentrations were approximately 50 µM and 2 µM, respectively, and loaded into thin walled 200 µL polypropylene microtubes (PCR tubes). This mixture was then irradiated for 1 h under 365 nm light at an intensity of 6.4 mW/cm^2^ from an LED source (M365LP1, Thor Labs) 14 cm away. Products were reduced using DTT solution (ThermoFisher) and separated on 4–12% BisTris PAGE gels (ThermoFisher) to observe photoconjugation. Full gel images are shown in Supplementary Fig. S2. Photocleavage was accomplished using the same irradiation setup. Photoconjugations to anti-FLAG antibody (anti-DYDDDDK clone 1557CT661.18.1, Lifespan Biosience LS‑C392574) or Cetuximab (Selleck Chemicals A2000) were done identically, with 100 µM PpL constructs that had been freshly purified. Photoconjugates were then purified from excess PpL using Amicon Ultra 50 kDa MWCO spin filter columns (Millipore-Sigma, UCFC505008). The unaltered antibody control conditions of our ELISA experiments, described below, validated Anti-FLAG binding to the FLAG peptide and Cetuximab binding to EGFR protein.

### Sortase mediated attachment of blocking peptides

Sortase A was expressed in *E.coli* using plasmid pET28a-SrtAdelta59 (Addgene #51138)^48^, and purified using Ni-NTA coated agarose beads (ThermoFisher). A photocleavable blocking peptide was synthesized (Biopeptide Inc.) with three N-terminal glycine residues followed by a photocleavable linker (Santa Cruz Biotechnology) and anti-FLAG or Cetuximab blocking peptide. Anti-FLAG or Cetuximab antibodies photo-conjugated with PpL fused to the Sortase recognition site, were diluted to 1 µM in TBS with 10 mM CaCl_2_ along with approximately 50 µM purified Sortase and 200 µM synthetic peptide. This was left to react overnight at room temperature, after which antibodies were purified using Amicon Ultra 50 kDa MWCO spin filter columns (Millipore-Sigma, UCFC505008).

### Protease cleavage

Chymotrypsin (Sigma Aldrich, C4129-250mg) was diluted to 200 µg/ml in PBS with 2 µM antibody conjugate and left to react for 1 h at room temperature. The protease reaction was then stopped with the addition of 1x Halt protease inhibitor cocktail (Fisher Scientific, 78430).

### Measuring relative binding affinity

Initially, ELISAs were used to test fundamental design features using the anti-FLAG antibody. For this, NeutrAvidin coated wells were bound with excess biotinylated FLAG epitope (Genscript), then blocked with PBST + BSA for 1 h. For Fig. 1c, 100 µl of PBST + BSA with 5 nM of anti-FLAG antibody and either FLAG epitope (Genscript) or PpL fused to FLAG epitope via the flexible linker shown in Fig. 1b were added at the indicated concentrations and incubated in the wells for 1 h. For Fig. 2f, anti-FLAG antibody alone, or anti-FLAG antibody covalently bound to PpL with linked photocleavable FLAG epitope, were irradiated for various amounts of time with 365 nm light, then diluted to 10 nM in PBST + BSA and added to wells in triplicate. For both figures, wells were then washed with PBST + BSA 3 times, and HRP-conjugated anti-Mouse secondary antibody was added in the same buffer to detect bound anti-FLAG, then developed with TMB substrate.

For Fig. 3, ELISAs were performed using NeutrAvidin coated plates with SuperBlock Blocking Buffer (Thermo# 15127). Each incubation step was allowed to proceed for 1 h at room temperature with shaking at 300 rpm. Between each incubation step the ELISA was washed by hand via Multichannel pipette with 200 µL of TBST three times. The plate was stamped out after washing to remove any remaining TBST before loading the next reagent. In Fig. 3, two columns for each antibody construct to be tested (cetuximab, cetuximab + construct or photoconjugated cetuximab + construct) had 100 µL of 0.1 nM Biotinylated EGFR (Acro Biosystems# EGR-H82E3) in TBST + 3% BSA loaded into each well. One column for each antibody to be tested was loaded with 100 µL of just TBST + 3% BSA as a non-specific binding control. The plate was then allowed to incubate for 1 h. A concentration curve of each antibody was prepared in TBST + 3% BSA. The curves had a starting concentration of 10 nM and were serial diluted 1:5. The plate was then washed, and 100 µL of each dilution were loaded into their three respective columns and allowed to incubate. The plate was washed and then 100 µL of Protein G-HRP (Invitrogen# 101223) diluted 1:5000 in TBST + 3% BSA was loaded into each well and allowed to incubate. The plate was washed again and 100 µL of 1-Step Ultra TMB (Thermo# 34029) was loaded into each well. After 10 min the reaction was quenched by adding 100 µL of 1 M H_2_SO_4_ to each well, and the absorbance at 450 nm was read using a Tecan Spark 20 M plate reader.

### Statistics and reproducibility

Statistical analysis was run using GraphPad Prism 9.2.0 for Fig. 2f using a Welch’s *t*-test (two-sided). For experiments in Fig. 3, for which statistical analysis was not conducted, general trends were reproducible, and but dependent on purification and storage conditions of the antibodies and antibody conjugates. At least two replicates for each assay using the same antibody preparation were performed, defined as repeating the entire ELISA assay including antigen coating, antibody or antibody conjugate binding, washing and antibody detection and development.

### Reporting summary

Further information on research design is available in the Nature Research Reporting Summary linked to this article.

## Supplementary information

Supplementary Information

Description of Additional Supplementary Data

Supplementary Data 1

Reporting Summary

## Data Availability

The datasets generated during and/or analyzed during the current study are available from the corresponding author on reasonable request. All data used in Fig. 2f and Fig. 3 is available in the file Supplemental Data 1.xlsx.
