# Supplementary material for: Turning antibodies off and on again using a covalently tethered blocking peptide
Source: Commun Biol. 2022 Dec 10;5:1357. doi: 10.1038/s42003-022-04094-1 (PMC9741643; doi:10.1038/s42003-022-04094-1)
Supplement: Supplementary file 1 — Supplementary Information [file 42003_2022_4094_MOESM1_ESM.pdf]

Supplementary Information:

TAATACGACTCACTATAGGGGAATTGTGAGCGGATAACAATTCCCCTCTAGAAATA
ATTTTGTTTAACTTTAAGAAGGAGATATACATATGGCTAGCGAGGTAACAATTAAGG
TGAACCTGATCTTTGCGGACGGAAAGATCCAGACGGCTGAATTTAAGGGAACTTTT
GAGGAGGCTACTGCAGAGGCCTACTAGTACGCGGATTTGCTTGCTAAGGTTAATG
GTGAGTACACTGCAGACCTGGAGGATGGTGGCAACCACATGAACATCAAATTCGC
CGGAGGTACCGAAGCGGCGGCTAAAGAAGCAGCAGCGAAGGAGGCGGCGGCAA
AAGAAGCCGCTGCAAAGGGAGGGAGTGAAGCGGCTGCGAAAGAGGCTGCCGCTA
AAGAAGCTGCAGCCAAGGAAGCTGCGGCTAAAGGAGGCAGTCTGCCAGAAACCG
GTGGTGGCCAAGGACAAAGCGGACAATGTATCAGCCCGCGTGGCTGCCCCGATG
GACCATACGTTATGTATAAGCTTGCGGCCGCACTCGAGCACCACCACCACCACCA
CTGAGATCCGGCTGCTAACAAAGCCCGAAAGGAAGCTGAGTTGGCTGCTGCCACC
GCTGAGCAATAACTAGCATAACCCCTTGGGGCCTCTAAACGGGTCTTGAGGGGTT
TTTTG

T7 promoter
T7 remnant tag
PpL C* with R33TAG mutation
TAG mutation site for BpA incorporation
$EAAAK_4$ Rigid linker
Sortase Site
EGFR blocking peptide
Remnant MCS
6xHIS tag
T7 Terminator sequence

Figure S1: Nucleotide sequence for Protein-L fused to flexible linker arm, sortase recognition site, EGFR blocking peptide and His-Tag. The different regions have been color coded with the legend below. The chymotrypsin cleavable linker was produced from a plasmid with identical sequence to this version, except that the sequence between the last rigid linker and EGFR blocking peptide was replaced with GGAGGCAGTGCCGCGCCTTTTGGTGGC.

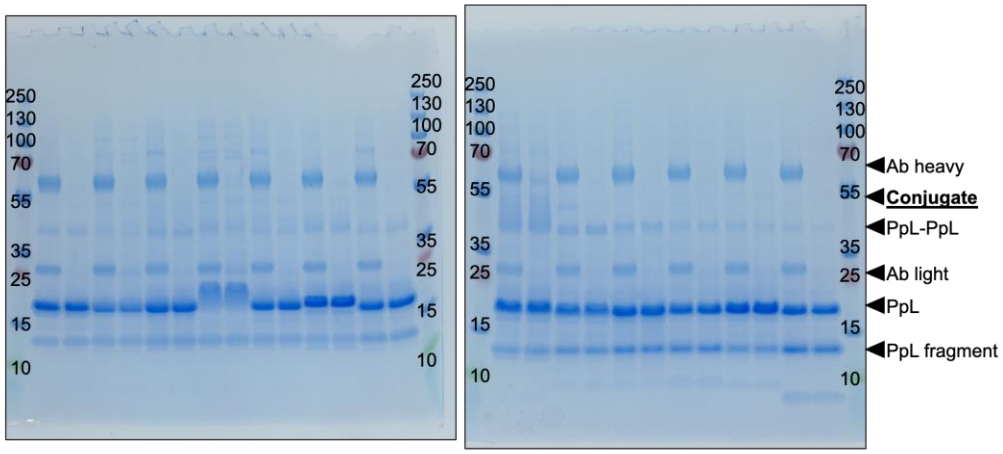

Figure S2: Full Gels of Figure 2B.

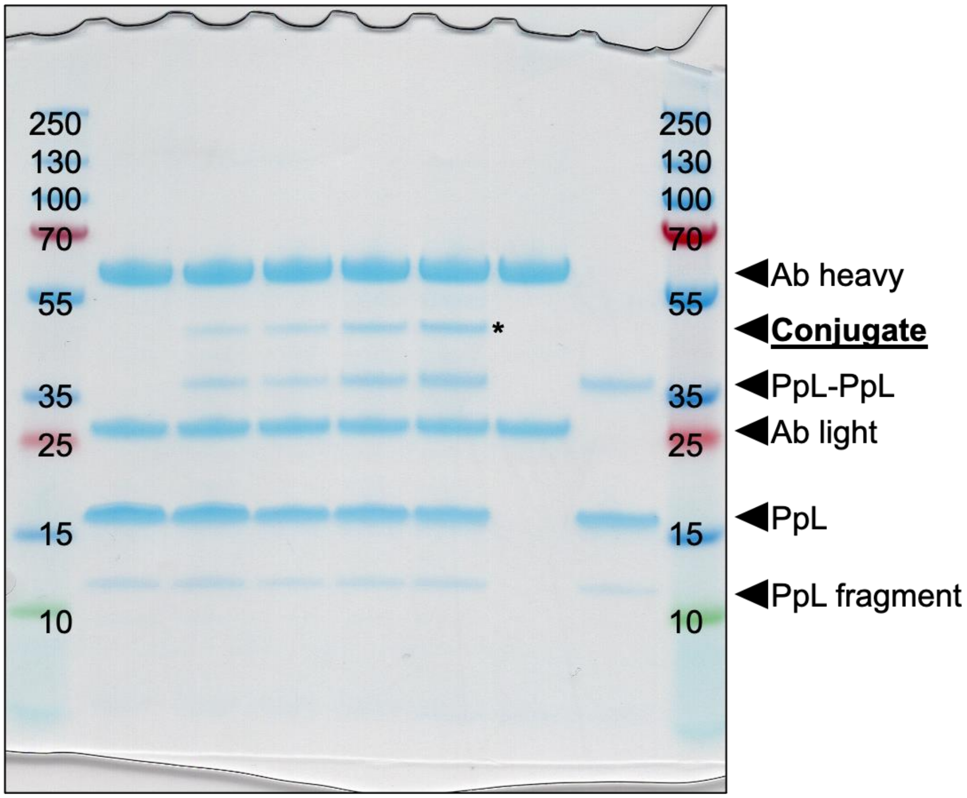

Figure S3: Full Gel of Figure 2D

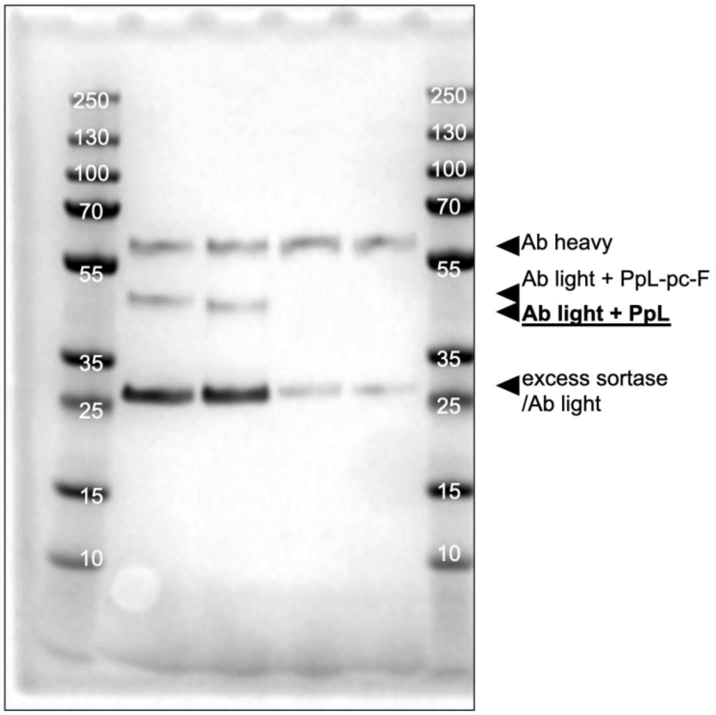

Figure S4: Full Gel of Figure 2E

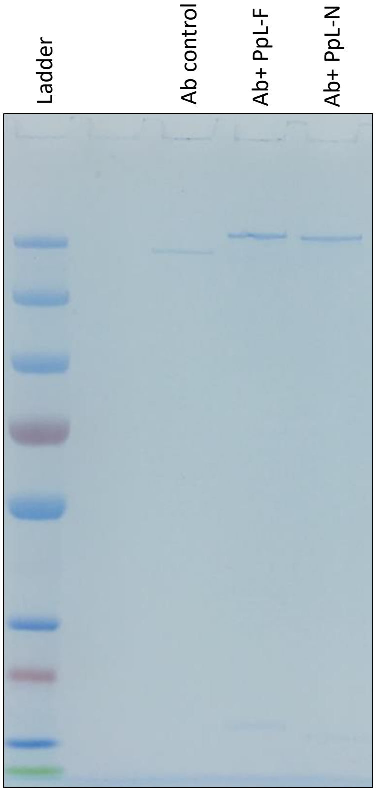

Figure S5: Campanion gel to Figure S4. Gel of anti-FLAG antibody photoconjugated to PpL tethered to anti-FLAG blocking peptide (PpL-F) or tethered to the sortase recognition sequence and no blocking peptide (PpL-N). Ab+PpL-N Photoconjugate was filtered to remove excess PpL and reacted with sortase to attach a photocleavable blocking peptide, then used in Figure 2F.

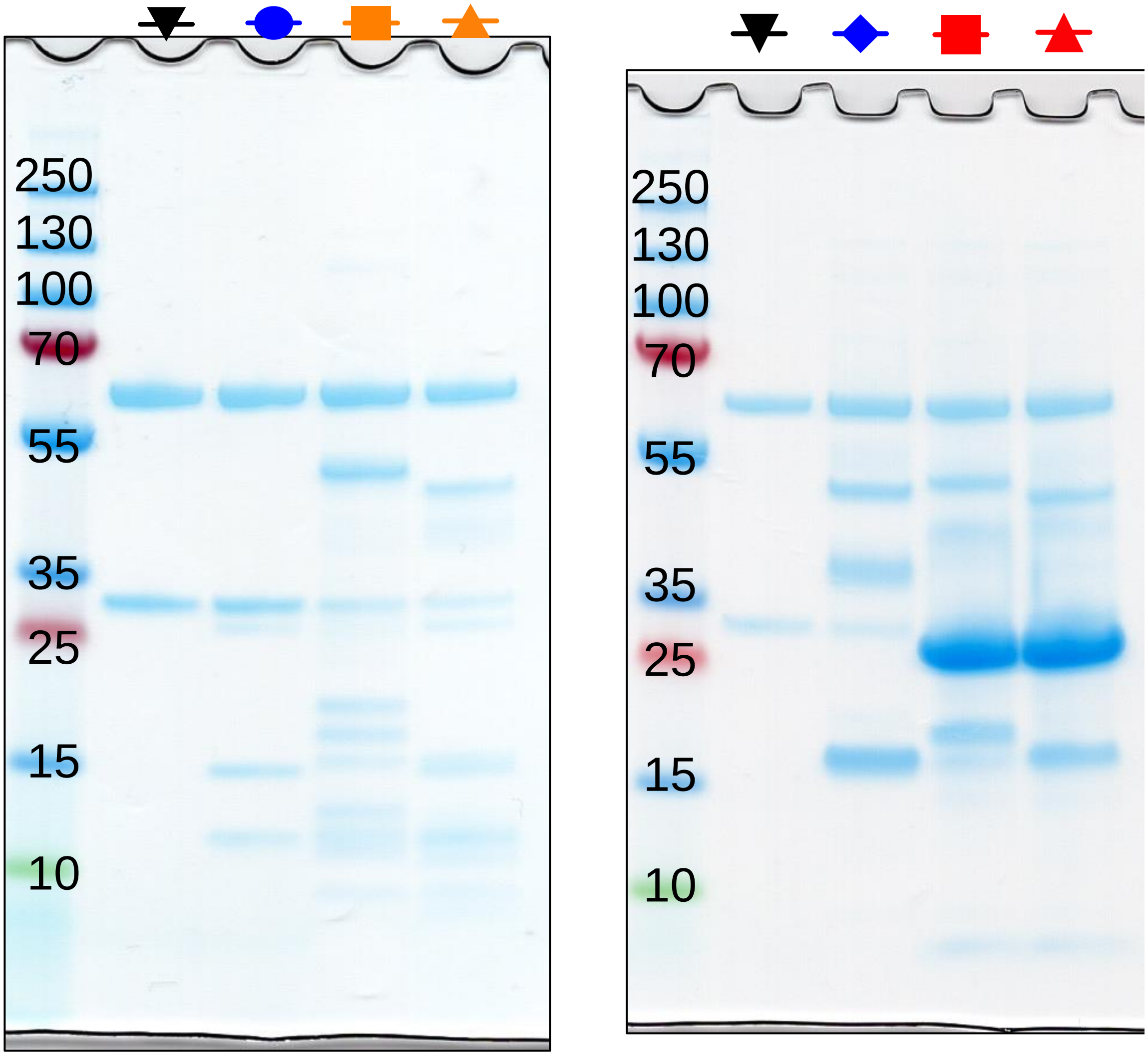

Figure S6: Full gels of figure 3C and figure 3D.
